# Epithelial extracellular vesicles induce inflammation and neutrophil activation in the *Pseudomonas aeruginosa* infected cystic fibrosis bronchial epithelium

**DOI:** 10.3389/fimmu.2025.1659951

**Published:** 2026-01-14

**Authors:** Meghan June Hirsch, Kuen-You Tsai, Luke I. Jones, Angela N. Morales, Hannah J. McIntire-Ray, Patrick H. Howze IV, Jarrod W. Barnes, Camilla Margaroli, Kristopher Genschmer, Stefanie Krick

**Affiliations:** 1 Department of Medicine, Division of Pulmonary, Allergy and Critical Care Medicine, Heersink School of Medicine, University of Alabama at Birmingham, Birmingham, AL, United States; 2 Gregory Fleming James Cystic Fibrosis Research Center, University of Alabama at Birmingham, Birmingham, AL, United States; 3 Department of Pathology, Division of Molecular and Cellular Pathology, Heersink School of Medicine, University of Alabama at Birmingham, Birmingham, AL, United States

**Keywords:** extracellular vesicles, cystic fibrosis, *Pseudomonas aeruginosa*, neutrophil activation, inflammation

## Abstract

**Introduction:**

Cystic fibrosis (CF) is an autosomal recessive disorder, which manifests in many organ systems including the lungs. Chronic inflammation is a hallmark of CF lung disease leading to bronchiectasis and lung function decline. This is worsened by airway colonization and recurrent infections due to opportunistic pathogens such as *Pseudomonas aeruginosa* [PA], but the crosstalk between host-bronchial epithelium and immune system has been under characterized. Extracellular vesicles have been found to mediate intercellular crosstalk in different lung diseases and EVs have been shown to be increased in the bronchoalveolar fluid of CF patients. We hypothesize that EVs from PA-infected CF bronchial epithelial cells can modulate pro-inflammatory cytokines and neutrophil migration and activation in an autocrine and paracrine manner.

**Methods:**

CF bronchial epithelial cells (CFBEs) and control bronchial epithelial cells (16HBEs) were infected with PA for 24 hours followed by EV isolation, which were used to treat uninfected CFBE and 16HBEs to assess expression and secretion of pro-inflammatory markers. In addition, the effects of EVs on neutrophil migration and activation were determined as well as the role of CFTR deficiency by using CFTR modulator therapy (Elexacaftor/Tezacaftor/Ivacaftor).

**Results:**

EVs derived from PA infected CFBEs (EVpPAs) increased IL-6, IL-8, and TNFα expression and neutrophil activation in CFBEs but not in 16HBEs. Interestingly, the effect of EVpPAs on inflammation was not attenuated by pre-treatment with ETI.

**Discussion:**

EVs from the PA-infected CF bronchial epithelium seem to facilitate an autocrine and paracrine pro-inflammatory response that is not attenuated by ETI treatment, suggesting a novel contribution of EVs to the chronic inflammatory phenotype observed in the PA-infected CF lung.

## 1 Introduction

Cystic fibrosis (CF) is an autosomal recessive disorder that effects about 1 in 4000 births in the U.S. with more than 150,000 people affected worldwide (1–3). CF is caused by mutations in the cystic fibrosis transmembrane conductance regulator gene (CFTR) which leads to muco-obstructive airway disease making this an ideal environment for opportunistic pathogens such as *Pseudomonas aeruginosa* (PA) (4–6). PA is the most common colonizing pathogen of people with CF (pwCF) greater than 35 years old (7). Colonization with PA is associated with an increase in pulmonary exacerbations, accelerated lung function decline and increased risk of mortality in pwCF (8–10). PA colonization and infection in the CF lung increases expression of the chemokine interleukin [IL] -8, which recruits neutrophils and cytokines (IL-1β, IL-6, and tumor necrosis factor (TNF) α), leading to further inflammation and immune cell responses (11, 12).

IL-8 is a cytokine well known to initiate neutrophil recruitment in the lungs especially in CF. CF neutrophils exhibit the GRIM phenotype named for their primary granule release, immunoregulatory function, and metabolic activities upon recruitment and transmigration to the CF lung tissue (13). These neutrophils express CD66b, which is a neutrophil specific surface protein that aids in adhesion to endothelial cells for transmigration, degranulation, and production of reactive oxygen species. Additionally, GRIM neutrophils downregulate surface expression of CD16 and increase primary granule release, measured by surface CD63 expression (13–15). Despite chronic neutrophilia in the CF lung, GRIM neutrophils cannot efficiently phagocytose bacteria, such as PA (15, 16). Therefore, chronic PA colonization and recurrent infections lead to a perpetuating cycle of chronic inflammation, chronic infection, and lung damage ultimately increasing mortality due to respiratory failure.

Improved nutrition and the use of inhaled antibiotics led to increased life expectancy in pwCF. The introduction of highly effective modulator therapy (HEMT), especially the restoration of CFTR activity using a triple combination of CFTR activators and potentiators (elexacaftor/tezacaftor/ivacaftor or ETI) led to further improvements in morbidity and mortality for patients, who are eligible by their CF genotype. Longitudinal studies though have shown that the bacterial burden with PA, while initially decreased on HEMT, increases again by two years post-HEMT initiation (6, 17). Additionally, some pwCF continue to be colonized even 3.5 years post initiation of ETI therapy (18). Therefore, more investigations are needed to identify the underlying mechanisms of PA-mediated lung injury in pwCF independent of their eligibility for HEMT. Recent reports show controversial results addressing consistent pan-anti-inflammatory effects of ETI and whether those are systemic or tissue specific (19–24). Further investigations are needed to determine the impact of ETI therapy on the inflammatory profile in the CF bronchial epithelium.

Extracellular vesicles (EVs), 30–1000 nm particles containing proteins, DNA, and several types of RNA, are produced through exocytosis from most cell types, including epithelial cells. They have been found in high abundance in bodily fluids (25, 26) and can mediate intercellular communication in many different inflammatory lung diseases including chronic obstructive pulmonary disease (COPD), asthma, interstitial lung disease and CF (25–29).

EVs have also been implicated in the disease pathology of CF. EVs have been found to be increasing in number in the bronchial alveolar lavage fluid (BALF) of people with CF compared to healthy controls and concurrently also increase in number with age (30, 31). EVs from CF bronchial epithelial cells demonstrate increased myeloperoxidase activity [MPO] and promote neutrophil migration (30). EVs isolated from corneal epithelial cells, infected with a PA lab strain (PAO1) induced cytokine production and neutrophil migration though using a different migration model (32). So far, there are not studies assessing prolonged PA infection on bronchial epithelial EV mediated autocrine and paracrine signaling and whether these effects can be attenuated by CFTR modulator therapy.

## 2 Results

### 2.1 EV isolation from bronchial epithelial cells

**Figure 1:**
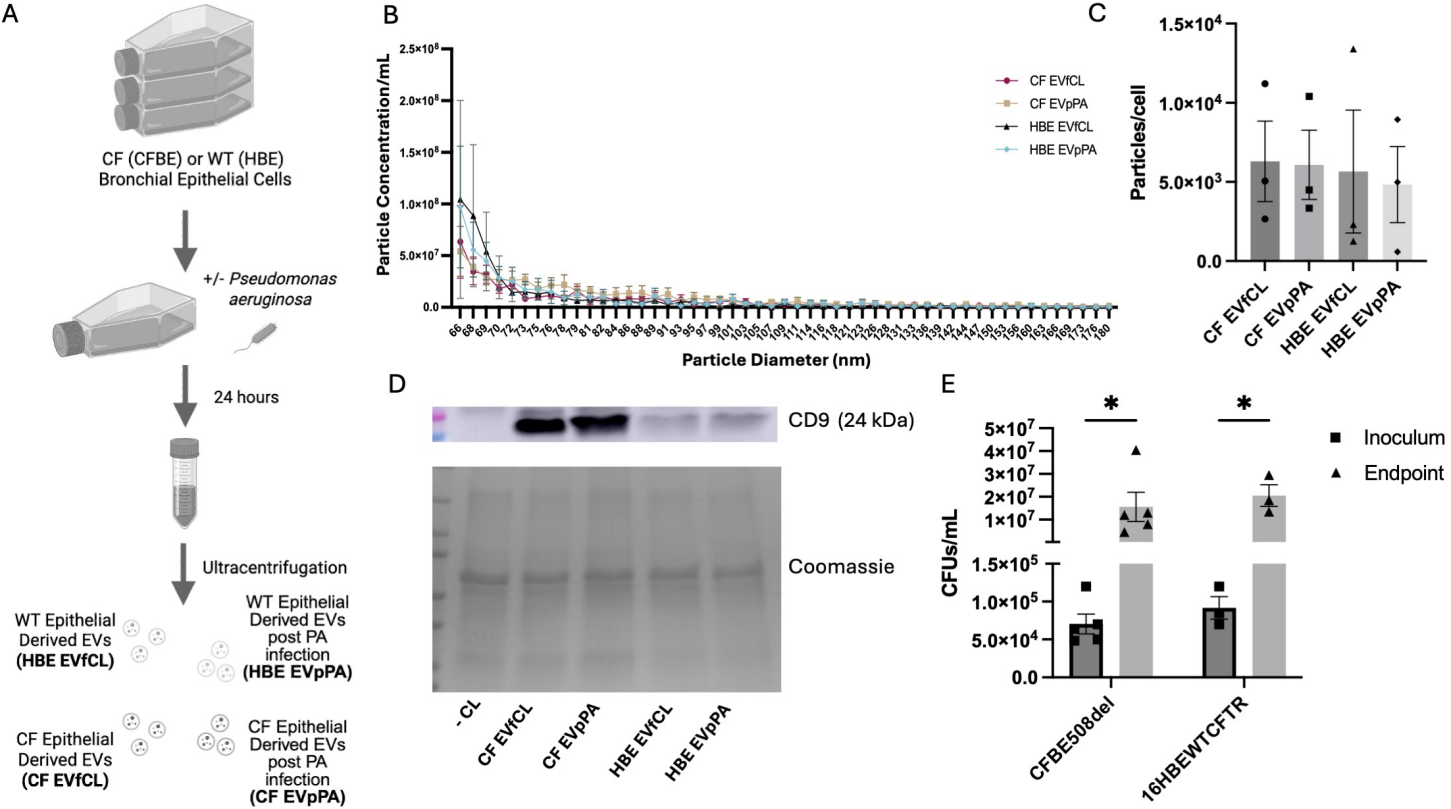
Isolation of bronchial epithelial extracellular vesicles. **(A)** Diagram depicting the different groups of EVs isolated from control and PA-infected bronchial epithelial cells. **(B)** Quantification of particle concentration per mL (y-axis) versus size analysis (x-axis) from CFBE and HBE EVs from control (EVfCL) and EVs post PA infection (EVpPA). **(C)** Quantification of particles per CFBE or HBE cell. **(D)** Representative western blots for CD9 and Coomassie stain from negative control, CF EVfCL, CF EVpPA, HBE EVfCL, and HBE EVpPA. **(E)** Quantification of inoculum and endpoint PA CFUs from PA-infected groups. These graphs represent n = 3–5 independent experiments. Statistical analysis was performed using a 1-way or 2-way ANOVA, followed by the Tukey’s multiple comparison or uncorrected Fisher’s LSD *post-hoc* tests, respectively. Data are expressed as mean ± standard error of the mean [SEM]. Differences were considered statistically significant if *p < 0.05.

Both CFBE and 16HBE cells were infected with PA for 24 hours and EVs were isolated from the media (**Figure 1A**). Isolated EVs had a size range of 65–400 nm with most particles ranging between 66–100 nm (**Figure 1B**). The number of particles/cell across the groups did not show any differences (**Figure 1C**). To properly classify the particles as EVs with an established EV marker (33), particles were analyzed for CD9 expression and compared to the media only control with Coomassie staining as a loading control. Our data showed that CD9 was present in all groups except the media only negative control but higher in the CFBEs, compared to 16HBEs (**Figure 1D**). PA burden was assessed for quality control at the time of infection (inoculum) and post infection (endpoint) indicating a significant increase in bacterial burden at the endpoint as expected but no difference between cell lines (**Figure 1E**).

### 2.2 EVs derived from PA-infected CFBEs increase neutrophil activation but not migration

**Figure 2:**
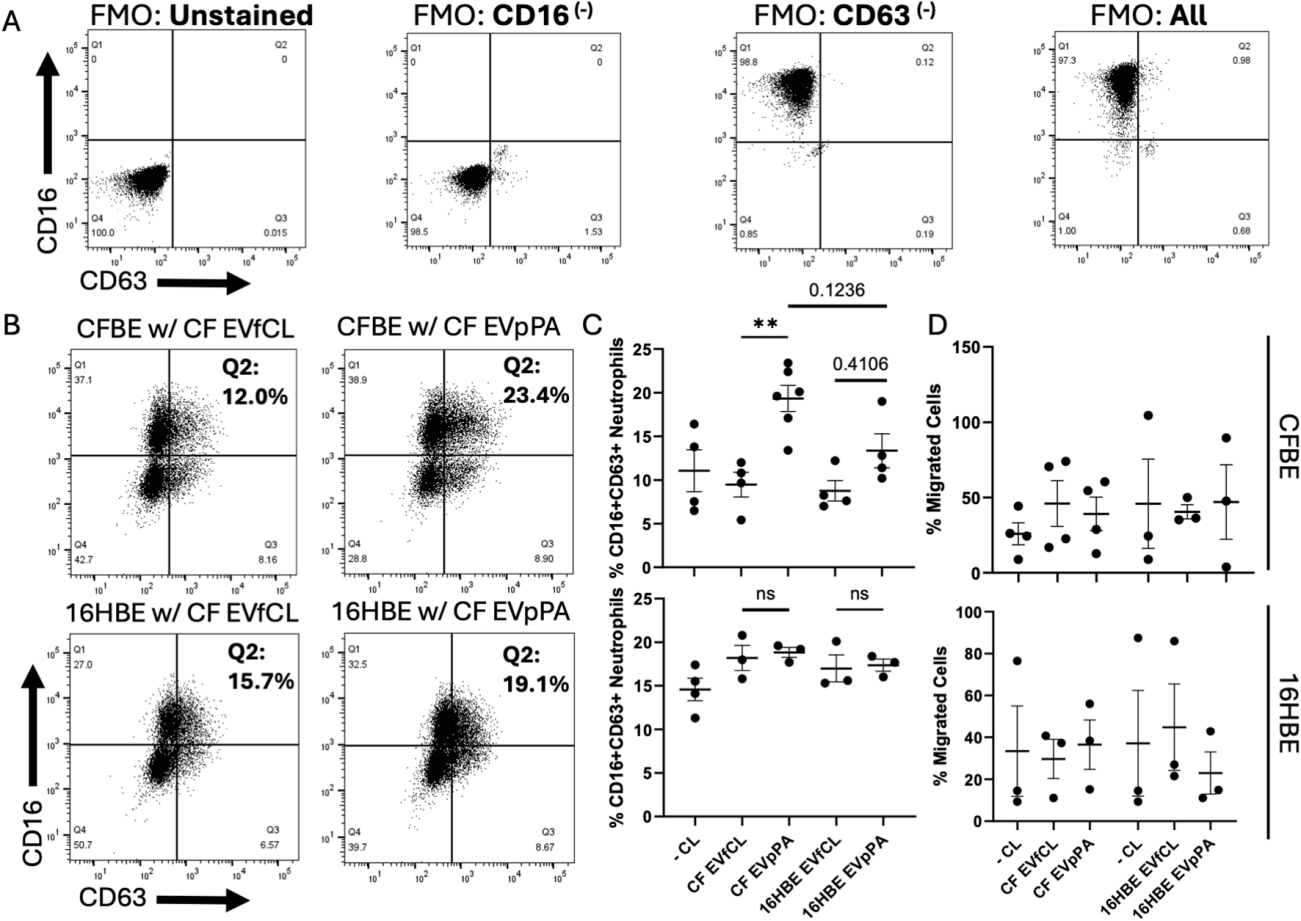
CF EVpPAs increase neutrophil activation but not migration. **(A)** Scatter plots with applied quadrant gates showing visualization of single live blood CD66b+ neutrophils with FMOs negative, CD16-, CD63-, and All when graphed by CD16 on the y-axis and CD63 on the x-axis. **(B)** Representative scatter plots with applied quadrant gates based on the blood neutrophil CD63 baseline from CFBE and HBE cells which were treated with the different EV groups. **(C)** Diagrams showing % of CD16+/CD63+ cells from the differentially treated neutrophil groups. **(D)** Diagrams showing % neutrophil migration from the differentially treated neutrophil groups. Statistical analysis was performed using a 1-way ANOVA, followed by the Tukey’s multiple comparison or unpaired t-test. These graphs represent n = 3–4 independent experiments. Abbreviation ns = no significance. Data are expressed as mean ± standard error of the mean [SEM]. Differences were considered statistically significant if **p < 0.01.

CFBEs and 16HBEs were infected with PA as shown previously (34). Isolated epithelial EVs were used together with leukotriene B_4_ (LTB_4_) to analyze their effects on neutrophil activation and migration using an adapted neutrophil transmigration model (14). To assess the GRIM activation status, we isolated neutrophils from healthy donors (**Supplementary Table 1**), gated on single live CD66b+ neutrophils before migration to obtain our FMOs (**Figure 2A**) and then single live CD66b+ neutrophils post transmigration to utilize expression of CD16 and CD63 (**Figure 2B**). CF EVpPAs significantly increased the percentage of CD16+CD63+ neutrophils after migration through CFBEs, when compared to “uninfected” CFBE-EVs (**Figure 2C**). This effect was not seen when neutrophils were stimulated in the same fashion but migrated through 16HBEs or when 16HBEs were used for EV isolation (**Figure 2C**). EV stimulation from any of the groups did not affect migration of neutrophils through CFBEs or 16HBEs (**Figure 2D**).

### 2.3 CF EVpPAs increase pro-inflammatory markers in the bronchial epithelium

**Figure 3:**
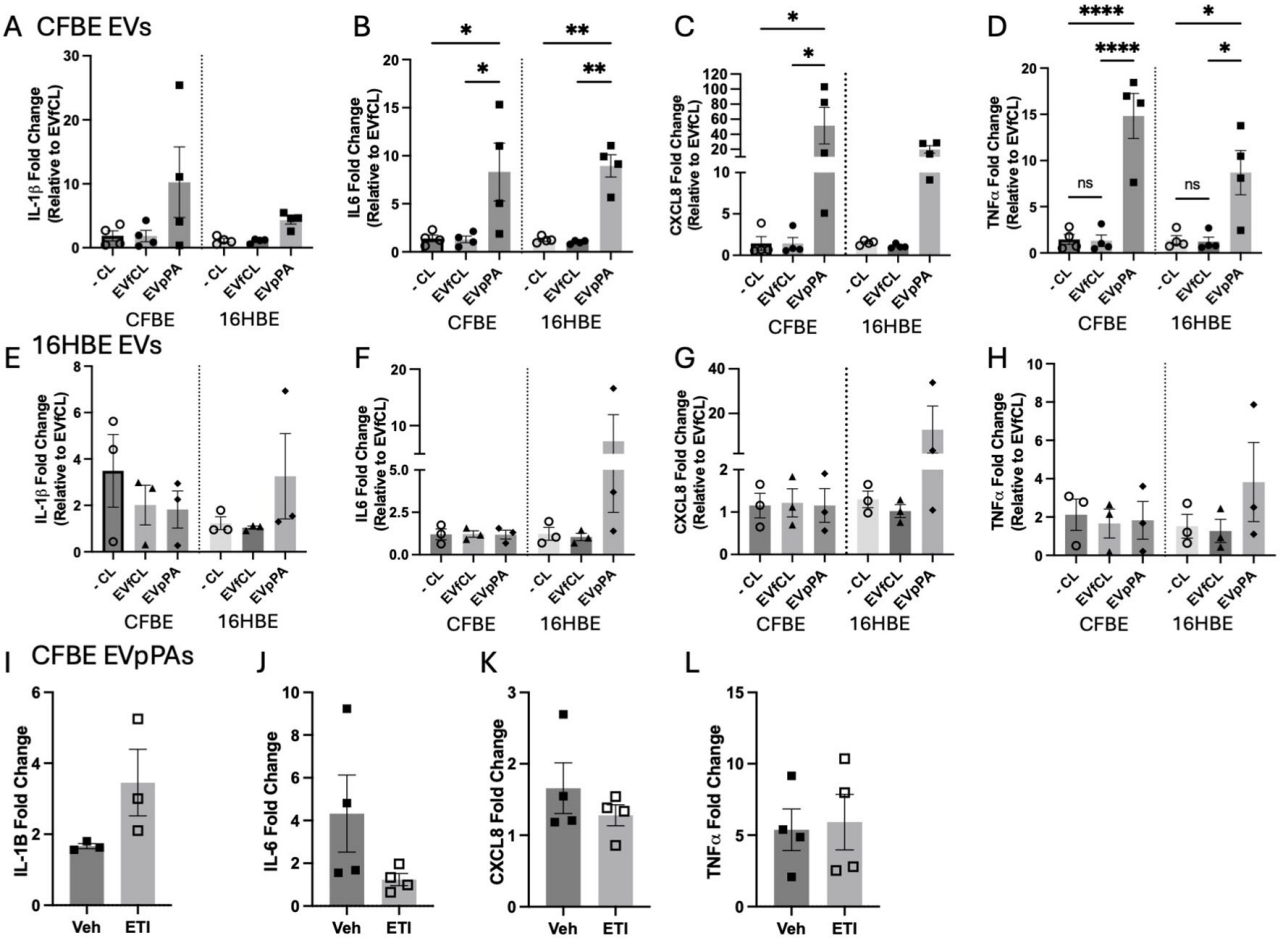
CF EVpPAs markedly increase pro-inflammatory markers in CFBE independent of CFTR correction. **(A)** Diagrams showing fold change levels of IL-1β mRNA, **(B)** IL-6 mRNA **(C)** CXCL8 mRNA and **(D)** TNFα mRNA fold change levels of CFBE and HBE cells treated with EVs from CFBEs either negative control, EVfCL, or EVpPA. **(E)** Quantification of IL-1β, **(F)** IL-6, **(G)** CXCL8 and **(H)** TNFα mRNA fold change levels of CFBE and HBE cells treated with EVs from HBEs either negative control, EVfCL, or EVpPA. These graphs represent n = 3–4 independent experiments. mRNA data was represented as fold change to untreated control. Statistical analysis was performed using a 1-way ANOVA, followed by the Tukey’s multiple comparison. Data are expressed as mean ± standard error of the mean [SEM]. Differences were considered statistically significant if *p < 0.05, **p < 0.01, ****p < 0.0001. **(I)** Graphs showing IL-1β, **(J)** IL-6, **(K)** CXCL8 and (**L)** TNFα mRNA fold change levels of CFBE41o- cells pre-treated ± ETI or vehicle (0.07% DMSO) followed by exposure to CFBE derived EVpPAs as described in previous figures. These graphs represent n = 3–4 independent experiments. Statistical analysis was performed using an un-paired t-test. Data are expressed as mean ± standard error of the mean [SEM]. Differences were considered statistically significant if *p < 0.05.

To assess potential autocrine effects of epithelial derived EVs, cells were first infected with PA, then, EVs were isolated from both 16HBEs and CFBEs (**Figure 1A**) and then used to treat uninfected 16HBEs and CFBEs. When CFBEs were treated with CFBE-derived EVpPAs, IL-6, CXCL8, and TNFα mRNA levels were significantly increased, and we observed a trending increase of IL-1β. We observed similar results when these CF-EVs were used to treat 16HBEs showing significant increases in IL-6, and TNFα with trending increases in IL-1β and CXCL8 (**Figures 3A–D**).

In contrast, treatment with 16HBE-derived EVs did not lead to upregulation of mRNA levels of these pro-inflammatory markers (**Figures 3E–H**). These data suggest that CF-derived EVs exhibit a pro-inflammatory phenotype that is not seen in 16HBE-derived EVs post-PA infection.

### 2.4 ETI treatment does not attenuate CF EVpPA induced inflammation

Since the EV-mediated pro-inflammatory response is more distinct in CFBEs and not mediated by EVs isolated from PA-infected 16HBEs, we wanted to test whether this effect is due to the intrinsic CFTR defect. CFBE41o- cells, which harbor the DF508 mutation and are therefore responsive to CFTR modulator therapy, were treated with ETI (VX-445 (1μM), VX-661 (3μM), VX-770 (3μM)) in minimum essential medium (MEM) + Ultroser G (USG) for 72 hours prior to EV treatment. Then, media was replaced with ETI + EVs (5 x 10^9^ EVs per well) and incubated for an additional 24 hours. ETI treatment, with the goal to restore CFTR function (35), did not change pro-inflammatory marker expression in EV-treated CFBEs, except a trend decrease in IL-6 and a trend increase in IL-1β (**Figures 3I–L**).

## 3 Discussion

**Figure 4:**
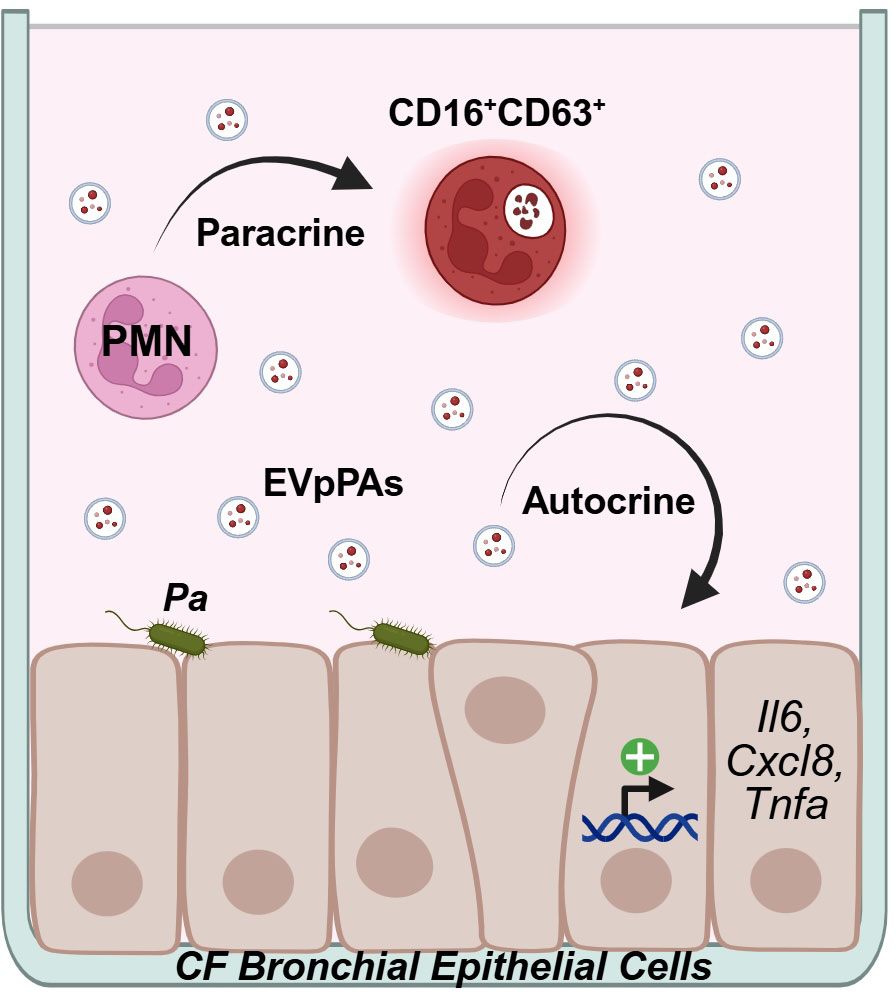
Diagram depicting autocrine and paracrine effects of bronchial epithelial EVs in the PA-infected CF lung.

Our study is one of the first studies to establish crosstalk between the bronchial epithelium and neutrophils in the PA-infected CF lung via EVs. We demonstrate that EVpPAs derived from CFBEs induce neutrophil activation and mediate an increase in pro-inflammatory markers via autocrine signaling, which persists despite CFTR correction with ETI (**Figure 4**).

We and others have shown previously that the CF bronchial epithelium expresses increased levels of pro-inflammatory markers such as IL-8, when compared to non-CF bronchial epithelial cells (36, 37). Furthermore, PA infection itself leads to further upregulation of IL-1β, IL-6 and IL-8 (12). Multiple reports have described the vicious cycle of infection and inflammation in the CF lung as one of the main contributors to lung injury (38). Several of the underlying PA virulence factors that are mechanistically responsible for the proinflammatory phenotype observed in CF include alginate and flagellin proteins (9, 39) and activation of host signaling pathways such as NF-κB (40). We have shown previously that PA infection can lead to an increase in the phosphorylation and resultant activation of PLCγ (12). It remains yet to be determined whether EV-induced inflammation is associated with the activation of canonical epithelial signaling mechanisms such as PLCγ.

One limitation of our study is that EVs were derived from a bronchial epithelial cell line, grown in submerged culture to establish our model. Future studies will need to be conducted in differentiated cultures and ultimately primary differentiated cultures, which could change outcomes (expression of pro-inflammatory markers and neutrophil activation) due to the role of differentiation and polarization on EV cargo and internalization.

In CF, neutrophilia is well described; however, neutrophils that are recruited to the airway display impaired phagocytosis of bacteria such as PA (8, 16, 41, 42). Furthermore, CF neutrophils possess an intrinsic granule-releasing, immunoregulatory and metabolically active (GRIM) phenotype with impaired bactericidal activity and adaptation to their environment resulting in a decreased bacterial clearance (15, 43, 44). In this study, primary granule release was significantly increased upon addition of EVs from CFBEs treated with PA compared to 16HBE controls, suggesting that response to PA may change the quality of EV cargo and in turn influence neutrophil activation. Further, the appearance of a CD16^low^/CD63^low^ neutrophil population in our *in vitro* model could be due to the use of different epithelial cells (i.e. large vs. small airways), which should be further investigated in future studies.

It has been shown that PA can increase primary granule release in airway neutrophils (15). PA itself can release outer membrane vesicles (OMVs), which have been shown previously to affect CFTR activity and can alleviate lung injury in a sepsis model (45, 46). It will remain to be studied in future investigations whether OMVs have a modulating effect in EV-mediated paracrine and endocrine crosstalk in our model. In the CF lung, EVs originating from neutrophils have been described as promoting the pro-inflammatory vicious cycle in CF airways via an autocrine loop leading activation of neutrophils via sustained inflammasome signaling (13), but to date, not many research studies have investigated EVs as potential vehicles for paracrine crosstalk, specifically between the CF bronchial epithelium and neutrophils. Koeppen et al. has shown that EVs from primary bronchial epithelial cells have a direct effect on PA via miRNA delivery reducing biofilm formation and increasing antibiotic sensitivity (47). The same group has also shown recently that HBEC EVs reduced PA burden and bronchoalveolar inflammation in a CF mouse model (48). Taken together, those findings suggest that EVs isolated from non-CF bronchial epithelial cells have a beneficial role in the PA-infected CF lung with direct effects towards the pathogen and the host.

Comparing our data, when we used HBE-derived EVs, they did not induce the pro-inflammatory and neutrophil activating response we observed with CFBE-derived EVs, which is consistent with the current literature. It was also surprising to see that CD9, which is a well-established marker for EVs, was expressed at much higher levels in EVs isolated from CFBEs, compared to 16HBEs, independent of PA infection. One possible explanation could be that CD9 is not only an exosome marker but also involved in growth, adhesion, signaling and inflammation (49) It will be of future interest to see further characterize differences in EV content between CF and control cells and whether CFBE-derived EVs exert a similar protective role in PA infection for both host and pathogen.

Lastly, the introduction of HEMT specifically ETI, has improved both quality of life and reduction of symptom burden for pwCF, especially respiratory symptoms, and some studies have shown that ETI may decrease pro-inflammatory markers such as IL-6, MMP9, and CXCL1 (50) while others show no systemic decrease with ETI therapy (51). Therefore, our finding that ETI pre-treatment of CFBEs does not attenuate EV-induced expression of pro-inflammatory markers fits within these previous reports. Veltman et al, suggested that persistent inflammation could be due to epigenetic changes, which are not reversed by CFTR correction (52). We did not observe significant differences in levels of IL-8 and TNFα between groups, which is consistent with previous reports from Allegretta et al. showing that stimulation with exoproducts of PA did not lead to significant differences in levels of IL-8 and TNFα between the control and ETI treated cells (53). The trending increase of IL-1β with ETI therapy could be an enhanced response to the inflammatory stimuli that the EVpPAs produce as suggested by Gentzsch et al, 2021 (54). Therefore, further studies are needed to fully elucidate the role of ETI therapy on pro-inflammatory signaling in the CF bronchial epithelium.

Overall, our work demonstrates that EVs isolated from the PA-infected bronchial epithelium potentially play a role in autocrine and paracrine crosstalk mediating a pro-inflammatory response and neutrophil activation. This pro-inflammatory response is not affected by ETI. Further studies are needed to decipher the underlying signaling pathways activated by EVpPAs and whether this mode of intercellular crosstalk can be therapeutically targeted.

## 4 Materials and methods

### 4.1 Human subjects, blood collection and neutrophil isolation

Blood was obtained from five healthy donors by venipuncture using K2-EDTA tubes. All samples were obtained with informed consent and the consents and protocol were approved by our Institutional Review Board (IRB-140414004) at the University of Alabama at Birmingham. People included in the study were “healthy” individuals (no listed or reported diseases) above the age of 18. Exclusion criteria included history of lung disease, history of smoking, known pregnancy, known diagnosis of autoimmune disease or immunocompromised state. Healthy donors had a mean age of 30.8 years of age, were 60% male and 40% female, and were 40% white, 20% Asian, and 40% Hispanic/Latino. Although these percentages were not fully representative in ethnicity of the CF population, these participants allowed us to generalize our findings across ethnic groups. Demographics are provided in **Supplementary Table 1.1**. Blood neutrophils were recovered as previously described using the density gradient Polymorph prep (Cosmo Bio USA) following the manufacturer protocol (15).

### 4.2 Cell culture and stimulation

The CFBEΔ508del (CFBE) and 16HBEWTCFTR (HBE) cell lines were obtained from the UAB CFRC Cell Model and Evaluation Core. The CFBE41o- cell line was a generous gift from Dr. Megan Kiedrowski. All CFBE and HBE cells were cultured as described previously (12). Briefly, cells were maintained in Minimum Essential Media (MEM) with the addition of 10% fetal bovine serum (FBS), 0.5% Pen-Strep, 1% L-glutamine, and 0.2% Plasmocin at 37°C with 5% CO_2_. 24 hours prior to PA infection or treatment with EVs, cells were washed with Clear MEM + 1% L-glutamine. After aspiration, the replacement media consisted of MEM with the addition of 2% Ultroser G (Crescent Chemical Company) and 1% L-glutamine, which was used throughout the rest of the experiment to avoid contamination with EVs contained in FBS.

### 4.3 Bacteria, culture and infection

The mucoid PA clinical isolate PAM57–15 was used for infection studies and cultured as previously described (12). Briefly, PAM57–15 was cultured in 5 mL of Luria Bertani broth overnight at 37°C. The PA infection protocol was modified for use in submerged cell culture (12). PA overnight cultures were washed and standardized to an inoculum of 7.5 X 10^4^ CFUs per mL, then inoculated in the supernatant of CFBE or 16HBE s cells and incubated for 24 hours prior to harvest for isolation of EVs.

### 4.4 EV isolation

Upon completion of the 24-hour PA infection of CFFBEs and HBEs, the supernatant was collected and spun at 3000 x g for 30 minutes, then transferred to a Beckman Coulter Ultracentrifuge and spun at 10,000 x g for 30 minutes and 120,000 x g for 2 hours, after which the supernatant was aspirated, and the pellet containing the particles was reconstituted in 1x PBS. Particles were counted using a Spectradyne nCS1. Particles from 65 nm to 400 nm were counted for the EV fraction and 5 x 10^9^ EVs were used for the described experiments. In addition, expression of CD9 via immunoblot was assessed as an established EV marker (23). Particles per cell were determined via the following formula: Average of (Particle Count/Total Cell Count Post Infection).

### 4.5 Immunoblotting

EVs were lysed using 1x RIPA with phosphatase inhibitor, phosphatase inhibitor cocktail II (RPI), and protease inhibitor, Roche cOmplete™ Protease Inhibitor cocktail (Millipore Sigma). Protein concentrations were determined by Bradford Assay. The protein concentrations were very similar, so loading was based on volume at the max volume of 50 μL per well. Coomassie gels were run to show equal protein loading. Anti-CD9 antibody was used as an established exosome marker. Gel electrophoresis and western blotting were performed as previously described (13). After electrophoresis, one gel was placed in GelCode™ Blue stain (ThermoFisher) overnight and then washed 3 times in DI water prior to colorimetric imaging for total protein. Membranes were blocked with 5% Bovine Serum Albumin (BSA) or 5% milk in Tris-buffered saline (pH 7.4) with 0.05% Tween 20 (TBST) for 30 mins and incubated overnight with the following primary antibody: mouse CD9 (Thermo Scientific) (1:1000). After 3 washes with 1x TBST, membranes were incubated with goat-anti-mouse peroxidase conjugated (Invitrogen) at 1:5000 in TBST for 45 mins. After 3–4 washes with 1x TBST, positive signals were visualized by chemiluminescence on an Amersham Imager 600 system (GE). Images were acquired using Image Lab Software (BioRad).

### 4.6 Neutrophil transmigration model

The neutrophil transmigration model was adapted from Dobosh et al. (15). Briefly, CFBEs and HBEs were seeded onto Alvetex filters at 2.5 x 10^5^ cells per scaffold with 2 mL of MEM 10% FBS, 0.5% Pen-Strep, 1% L-glutamine, and 0.2% Plasmocin. The second day post seeding cells were placed at the air-liquid-interface (ALI) and the basolateral media was replaced with MEM containing glutamine (1%), Pen/Strep (0.5%) and 2% USG and maintained 14–19 days to allow for polarization and then filters were flipped and incubated with freshly isolated neutrophils on the basolateral (to apical) side for 12 hours (13, 44, 55). Leukotriene B4 (LTB4) 100 nM was added basolateral as a stimulus for neutrophils to transmigrate, while the EVs were added basolateral as well.

### 4.7 Flow cytometry

Neutrophils were collected from the media post transmigration, counted and stained with surface markers and dyes as previously described (56): CD66b, CD63, CD16, and Live/dead zombie dye (Biolegend). Flow cytometry data were acquired on a FACSymphony A3 (BD Biosciences) and results were analyzed by FlowJo v.10.10.0.

### 4.8 RNA isolation, cDNA synthesis, and quantitative real time PCR

Total RNA was isolated from cells as previously described (12). RT-qPCR was performed on an Applied Biosystems StepOnePlus using the following TaqMan probes: Interleukin-8 (Hs00174103_m1, CXCL8), Interleukin-6 (Hs00174131_m1, IL-6), Interleukin 1-β (Hs01555410_m1, IL-1β), TNFα (Hs00174128_m1, TNF) and reference gene glyceraldehyde-3-phosphate dehydrogenase (GAPDH). Fold change was calculated via the ΔΔCT method previously described (12).

### 4.9 Quantification and statistical analysis

Data were analyzed using GraphPad Prism 9 for Macintosh (GraphPad Software). Unpaired t-test, One-way or Two-way ANOVAs were performed, followed by Tukey’s multiple comparisons or uncorrected Fisher’s LSD post-doc test using a 95% confidence interval where indicated. All graphs represent at least n = 3 independent experiments. Data are expressed as means ± standard error of mean [SEM]. Differences between groups were considered statistically significant if P < 0.05.

## Data Availability

The original contributions presented in the study are included in the article/**Supplementary Material**. Further inquiries can be directed to the corresponding author.
