# Supplementary material for: Epithelial extracellular vesicles induce inflammation and neutrophil activation in the Pseudomonas aeruginosa infected cystic fibrosis bronchial epithelium
Source: Front Immunol. 2026 Jan 14;16:1659951. doi: 10.3389/fimmu.2025.1659951 (PMC12846939; doi:10.3389/fimmu.2025.1659951)
Supplement: Supplementary file 2 [file Table1.docx]

Supplementary Material

## Supplementary Tables

| Demographics for Neutrophil Donors | | |
| --- | --- | --- |
| Age: Mean (Range) | 30.8 | (22-47) |
| Gender: Male/Female | 60%/40% | (3/2) |
| Ethnicity: |  |  |
| White | 40% | (2/5) |
| Asian | 20% | (1/5) |
| Hispanic/Latino | 40% | (2/5) |

**Supplementary Table 1.** Demographics for Healthy Neutrophil Donors for Transmigration and EV treatment.

## Supplementary Figures

**Supplementary Figure 1.** Representative Flow Cytometry Gating Strategy for Neutrophils Transmigrated through CFBE or HBEs.
