# Supplementary figures and images for: Epithelial extracellular vesicles induce inflammation and neutrophil activation in the Pseudomonas aeruginosa infected cystic fibrosis bronchial epithelium
Source: Front Immunol. 2026 Jan 14;16:1659951. doi: 10.3389/fimmu.2025.1659951 (PMC12846939; doi:10.3389/fimmu.2025.1659951)

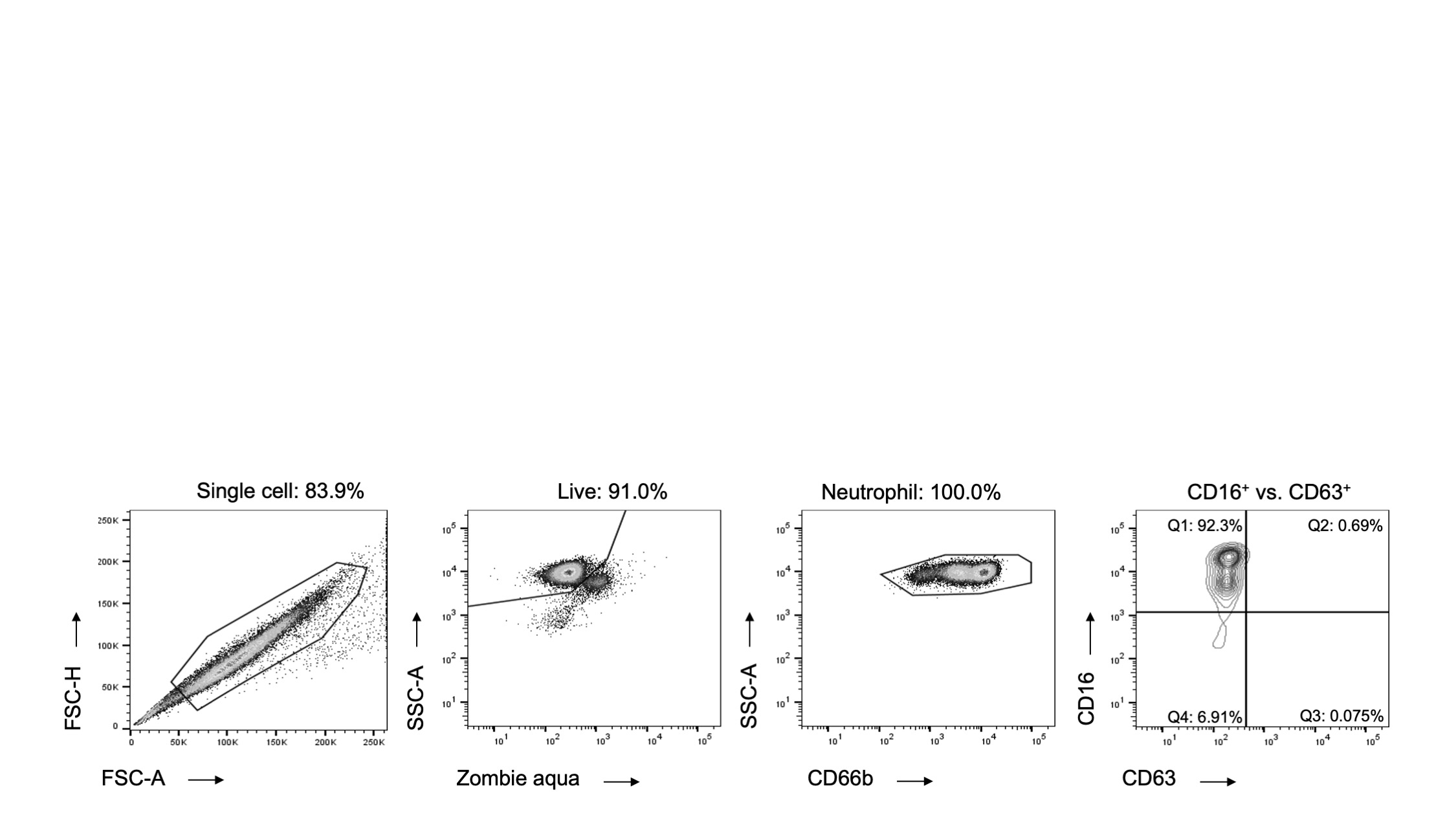

Supplement: Supplementary file 3 [file Image1.jpeg]
